# Variability of Essential and Nonessential Fatty Acids of Irish Rapeseed Oils as an Indicator of Nutritional Quality

**DOI:** 10.1155/2022/7934565

**Published:** 2022-01-12

**Authors:** Rebecca Coughlan, Siobhan Moane, Tracey Larkin

**Affiliations:** ^1^FooDs@ LIT, Technological University of the Shannon, Midlands Midwest, Ireland; ^2^Shannon Applied Biotechnology Centre, Technological University of the Shannon, Midlands Midwest, Ireland; ^3^Department of Applied Science, Technological University of the Shannon, Midlands Midwest, Ireland

## Abstract

The low saturated fatty acid content of rapeseed oil has resulted in it being classed as one of the most health-benefiting culinary oils. This study determines whether Irish rapeseed oils contain identical fatty acid profiles or whether distinct profiles exist between producers and producers' successive oil batches. The fatty acid content of Irish rapeseed oils was determined in terms of the desirable MUFA and PUFA and saturated content of these oils. The fatty acid composition demonstrated significant differences in individual unsaturated fatty acid content, while total saturation had insignificant differences. Saturated fatty acid content ranged from 6.10 to 15.8%, while unsaturated fatty acids ranged from 84.20 to 90.10%. Moreover, individual fatty acid content exhibited significant differences (*p* < 0.05). Oleic acid (C18:1), linoleic acid (C18:2), and stearic acid (C18:0) contents were considered significantly different from other fatty acids detected. The third successive batch from each producer exhibited lower oleic acid content, and the third batch contained higher linoleic acid content, at the same time maintaining a desirable unsaturated fatty acid composition. Studies suggest that differences in the fatty acid composition may be due to cultivation practices such as climate, soil composition, sowing and harvesting, processing techniques, and oxidation reactions.

## 1. Introduction

Lipids are a fundamental source of nutrition directly influencing health [1]. Culinary oils composed of saturated fatty acids (SFA) give rise to low-density lipoproteins (LDLs), elevating cholesterol levels and leading to coronary diseases. In comparison, the unsaturated fatty acid (USFA) portion has lower cholesterol levels and is essential [1]. Many epidemiological studies emphasise the nutritional benefits of USFA, with a strong focus on the necessity of polyunsaturated fatty acids (PUFA) as they are not synthesised in the body and must be obtained directly from food [2]. Culinary oils that contain higher levels of PUFA can be considered to have a higher nutritional value. In Ireland, coronary heart disease (CHD) is one of the highest causes of death due to inadequate diet; therefore, demands for health benefiting oils such as rapeseed oil have increased [3].

Rapeseed oil is the second leading culinary oil produced globally as it contains a desirable fatty acid composition of predominantly unsaturated fatty acids and a healthy bioactive compound content [4]. Studies investigating the fatty acid composition of rapeseed oils suggest the total USFA portion can range from 92.2% to 93.7%, with a higher PUFA content than other culinary oils [5–7]. Oleic acid (C18:1) is reported in numerous publications for its beneficial activity on LDL cholesterol [8]. The fatty acid profile of rapeseed oils has been shown to contain higher oleic acid (C18:1) and linoleic acid (C18:2) levels than other fatty acids, indicating that rapeseed oil is of high nutritional value [9]. Studies have proven that fatty acids are an essential part of the daily dietary requirement due to their significant biological function [2]. Many epidemiology studies have focused on the nutritional benefits of unsaturated fatty acids, with a strong focus on PUFAs, such as linoleic acid and linolenic acid, as they must be obtained directly from food [1].

To maintain oil quality after processing, the fresh bottle samples require correct storage conditions. Oil oxidation is problematic, reducing the oil quality over the storage period due to the breakdown of essential fatty acids, resulting in increased free fatty acids, resulting in oil rancidity, reducing the nutritional benefits significantly [10]. Gómez-Alonso et al. (2007) and Kiralan and Ramadan state that oil quality maintains better in dark and cool conditions for 12 months than storage at ambient temperature. Wroniak and Rękas reported similar results concerning peroxide value and total phenolic content but reported no changes in the fatty acid composition of closed rapeseed oils stored at 4°C for 12 months storage [5]. Moreover, it was noted that packing material does not affect the quality of the oil in terms of fatty acid composition or oxidative stability but the availability of oxygen to mix and increase free space within the oil deteriorates the oil during use and storage periods [4, 5].

Ireland imports many culinary oils annually; in 2017, Ireland imported 131,000 tons of olive oil. However, Zahoor and Forristal suggest that Ireland could produce more of its culinary oil due to its agricultural background and ideal climatic to cultivate rapeseed as part of annual crop production [11]. This can be financially beneficial to the economy and agricultural sector while also making nutritional and healthy benefiting culinary oils for consumers. The quality of an oil is directly influenced by the cultivar, cultivation, harvesting, and processing techniques. An oil of high quality contains nutritional benefits such as a high USFA content and high antioxidant capacity [12, 13]. For many years, culinary oils have been studied to profile the nutritional attributes oils contribute to consumers. The fatty acid profile of oil is directly related to the nutritional quality an oil possesses. Many studies have comprehensively characterised the nutritional potential of rapeseed oil based on essential fatty acid composition [14–17]. If the Irish rapeseed oils being studied here demonstrate abundance in essential fatty acid content, the nutritional quality of rapeseed oil from Ireland can be considered a significant factor in producing Irish rapeseed oils.

Routinely fatty acid determination has been carried out using gas chromatography mass spectroscopy (GCMS) techniques. However, these methods require the derivisation and alteration of samples. Bromke et al. found LCMS detection of fatty acids comparable to GCMS, where samples only needed to be filtered before injection. Besides, LCMS allowed for the differentiation between isomers and the separation of fatty acids as large as 26 carbons in length. It was noted that while the precision and correlation with standard peaks tend to be higher for GCMS, peak detection and annotation does not require standards for LCMS detection. Several studies have implemented LCMS for fatty acid detection [18].

While many studies have been conducted characterising culinary rapeseed oils' fatty acid composition, no comprehensive research has been undertaken on those produced in Ireland. The composition of the fatty acids in the oil can directly impact consumers' health [14]. Therefore, the fatty acid composition is a valuable guide to highlight the potential benefits of culinary oil for consumer consumption. Particularly in SFA and total USFA and PUFA content, highlighting the least and most desirable components of an oil's fatty acid composition from a consumer's perspective.

This study aims to conduct a fatty acid analysis of Irish rapeseed oils from 6 Irish producers, focused on the total fatty acid content and the individual fatty acid profile from each rapeseed oil sample. This will determine whether significant differences between culinary rapeseed oils from different Irish producers exist. Additionally, differences within successive batches from each producer may indicate variance with the consistency of the fatty acid content of the rapeseed oils produced from period to period.

## 2. Materials and Methods

### 2.1. Samples

Commercially available samples of cold-pressed Irish rapeseed oils were selected from six Irish rapeseed producers available in local supermarkets and were purchased for the study. Samples were randomly assigned a number 1 to 6 with the specific code PRO1–PRO6, while each “batch” of the samples was coded B1–B3. Individual batches were determined by different processing dates on each bottle and ranked based on the processing date, i.e., batch 1 had the earliest processing date printed.

The coding system was as follows: PRO1B1 represents the rapeseed oil batch produced at the earliest point of the 3 samples by producer 1. Each “batch” was evaluated in triplicate where *n* = 18 samples in total.

### 2.2. Sample Preparation and LCMS Conditions

Fatty acid profiling was carried out based on the method outlined by Otero et al. with slight modifications [19]. This modification included pure samples rather than extracted lipids as the oils are primarily composed of lipids. Each oil sample was diluted 20 times in a mixture of methanol and dichloromethane (2 : 1), and 10 *μ*L of the diluted sample was injected into the HPLC system equipped with a Q-TOF mass spectrometer (Agilent 6520 series), in triplicate for analysis [19]. Samples were resolved by an Agilent C-18 Poroshell 120 column (2.7 *μ*m, 3.0 × 150 mm) separation analytical column. The mobile phase used was a gradient elution, and mobile phase A consisted of ultrapure water and 2 mM ammonium acetate. Mobile phase B consisted of a 2 mM ammonium acetate mixture in 95% acetonitrile. The MS was operated in a negative ionisation mode, scanning from 50 to 1,100 m*/z*. The fragmentor voltages were kept at 175 and 65 V, respectively. Drying gas flow rate, temperature, and nebuliser pressure were at 5 L min^−1^, 325°C, and 0.21 MPa, respectively. Peak evaluation and sample screening were conducted using Agilent Mass Hunter Qualitative analysis Navigator software version B.08.00. Standard fatty acids such as palmitic acid (C16:0) and oleic acid (C18:1n-9 *cis*) were used to validate the LC-MS method for fatty acids analysis by comparing their retention time (RT) and specific, accurate mass (Sigma, Ireland). All fatty acids in the samples were then identified based on their known accurate mass, and their relative content was recorded as per their peak area. The peak area of each fatty acid was the average of triplicate samples.

## 3. Results and Discussion

### 3.1. Data Analysis

**Figure 1:**
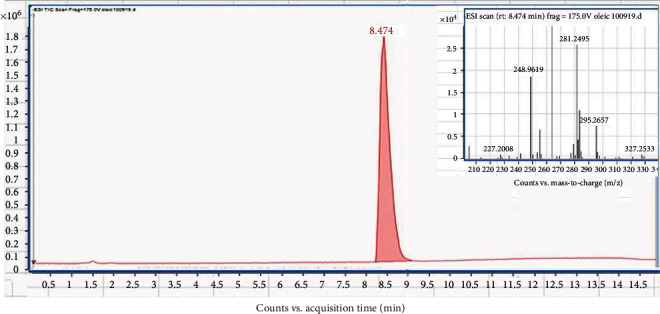
Representative ESI TIC peak and corresponding mass spectra (inset image) of standard oleic acid (C18:1) showing characteristic m/*z* value (281.249) and retention time (8.47 min).

Samples of cold-pressed rapeseed oil from Irish producers were analysed for fatty acid composition. Each sample was injected in triplicate, and the mean peak area for each fatty acid was used to generate the peak percentage for each fatty acid. Known standards were used as guidelines for retention time and m/*z* comparison for the fatty acid in the oil samples.  Figure 1 illustrates the oleic acid (C18:1) standard used at 10 *μ*g/mL concentration. The chromatogram contains a single peak at approximately 8.47 minutes. The chromatogram's inset depicts the parent ion for oleic acid (C18:1) with an m/*z* value of 281.249 in negative ionisation mode, which matched the library as the m/*z* of oleic acid (C18:1) is 282.249.

Likewise, the m/*z* of peaks detected in samples were compared to a library of 38 known fatty acids ranging from 8 to 24 carbons chains and varying in saturation.  Table 1 outlines the theoretical m/*z* to the experimental m/*z* detected of the individual fatty acids [19].

**Figure 2:**
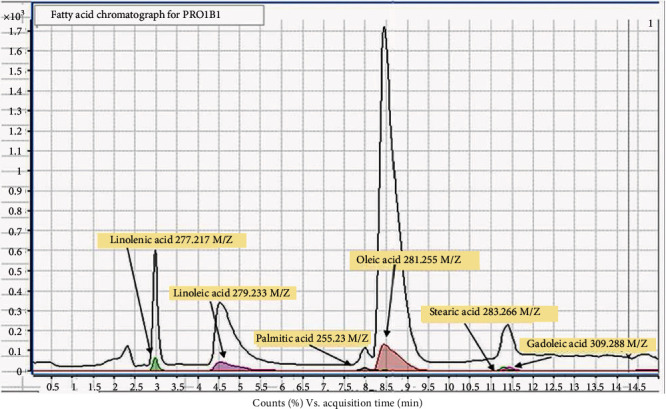
Green: linolenic acid (C18:3); purple: linoleic acid (C18:2); grey: palmitic acid (C16:0); red: oleic acid (C18:1); lime: stearic acid (C18:0); magenta: gadoleic acid (C20:0). Separating individual fatty acids in the rapeseed oil from producer 1, batch 1, via LCMS as outlined in Materials and Methods. Coloured peaks represent overlayed chromatograms of identified individual fatty acids separated and detected in PRO1B1.

A positive match to the library was a minimum match of three significant figures to parent ions of the library values. Similar retention time to the standards was also considered with allowances for matrix interference. Each sample was analysed in triplicate, and mean values were used to calculate the percentage of each fatty acid present within the identified profile of fatty acids (a combination of all fatty acids detected =100%).  Figure 2 outlines the separation of fatty acids in a representative sample PRO1B1, where the abundant fatty acids are highlighted in the inset of the chromatogram.

Parent ions and retention times of the fatty acids in the rapeseed oil samples were comparable to the oleic acid (C18:1) standard in Figure 1.

### 3.2. Fatty Acid Composition of Irish Rapeseed Oils

Fatty acid profiling was executed in triplicate for three batches of rapeseed oil per Irish producer (*n* = 54).  Table 2 outlines the dominant compositional fatty acids for each rapeseed oil tested. The total SFAs and USFAs were calculated for each batch.

Fatty acids were detected and reported in terms of the percentage of fatty acid per sample. When combined, each fatty acid percentage yields the total identifiable fatty acid content (100%). The fatty acid composition of Irish rapeseed oils tested comprised a mixture of saturated and unsaturated fatty acids. Palmitic acid (C16:0), stearic acid (C18:0), oleic acid (C18:1), linoleic acid (C18:2), linolenic acid (C18:3), and gadoleic acid (C20:0) were the most predominant fatty acids composing each rapeseed oil profile. Traces of other fatty acids make up a smaller proportion of each oil. The trace fatty acids range from 1.40% to 5.30%, indicating the primary compositional fatty acids construct 94.70% to 98.60% of the total profile.

### 3.3. Saturated Fatty Acids

The SFA content of the Irish rapeseed oils ranged from 6.1% to 15.8%, where the rapeseed oil from producer 2, B2, had the lowest SFA content, and the rapeseed oil from producer 4, B1, had a much higher SFA composition (Table 2). Many studies have reported the SFA (%) content of rapeseed oils ranging from 6.14% to 9.48% [7, 14, 20, 21]. Five of the 6 Irish rapeseed oils tested had a total SFA content agreeing with these reported values. In contrast, the rapeseed oil from producer 4, B1, contained a significantly higher SFA content (*p* < 0.05).

Palmitic acid (C16:0) and stearic acid (C18:0) are the most abundant SFA in the rapeseed oils tested. Palmitic acid (C16:0) content is more significant than stearic acid (C18:0), which corresponds with the data reported by Frančáková et al. and Potočnik et al. The palmitic acid (C16:0) content of rapeseed oils tested ranged from 2.21% to 7.99% [7, 22, 23]. The rapeseed oil from producer 2, B2, had the lowest palmitic acid (C16:0) content, and the rapeseed oil from producer 4, B1, had the highest. ANOVA analysis determines the difference in palmitic acid (C16:0) be considered statistically significant (*p* ≤ 0.05). Stearic acid (C18:0) content ranged from 1.48% to 4.34%. The rapeseed oil from producer 6, B2, had the highest content, and the rapeseed oil produced by producer 3, B1, had the lowest content of stearic acid (C18:0). These differences, too, was deemed statistically significant (where *p* ≤ 0.05). These findings agree with data from various literature that reported palmitic acid (C16:0) content in rapeseed oil varying from 4.06% to 5.20%, and stearic acid (C18:0) was ranging from 1.31% to 4.40% [7, 14, 22, 24].

Increased SFA similar to those observed with PRO4B1 can arise at various production stages. Szczepanek et al. and Onemli stated that the organic matter in the soil might influence the fatty acids in the final oil content, specifically, individual fatty acids such as palmitic acid (C16:0) [25, 26]. Joughi et al. determined that late sowing of seeds can increase the SFA proportion of the oil content by increasing palmitic acid (C16:0) and stearic acid (C18:0) [27]. Moisture content influences free fatty acid production, but humidity during storage can also lead to further lipase production, increase the free fatty acids, and reduce the oil's stability. Additionally, environmental factors during cultivation, such as heat and light intensity, are essential in fatty acid development, mainly oleic acid (C18:1) and palmitic acid (C16:0) [28, 29]. Any of these factors may explain the variations in palmitic and stearic acid (C18:0) concentrations observed in this study. Furthermore, it can be assumed the rapeseed crop from which these oils were produced was cultivated in different geographical regions in Ireland, which can vary in soil type, weathering, and altitude. These differences may affect the development of rapeseed crops and oil fatty acid content and quality.

SFA are undesirable fatty acids associated with increased cholesterol in the blood. Literature has found that a specific cholesterol ratio to HDL in the bloodstream is a more precise heart disease indicator than just LDL concentration [30]. This study also stated that individual fatty acids could affect the ratio. Fatty acids such as myristic and lauric can cause plasma increases, whereas palmitic and stearic acid (C18:0) only slightly influence the ratio. Thus, if the SFA content of rapeseed oil is composed primarily of palmitic acid (C16:0) and stearic acid (C18:0), it can be assumed the effect of rapeseed oil on the cholesterol to HDL ratio would be minimal, if at all. Additionally, Behenic acid is presented in a minor amount in rapeseed oil (0.37%) and has been detected in low quantities in some Irish rapeseed oils. Konuskan et al. state that due to the little bioavailability of behenic acid due to its long-chain structure, it would cause less effect on cholesterol content [6].

### 3.4. Unsaturated Fatty Acids

The USFA content of rapeseed oils accounts for approximately 90% of the fatty acid composition [22, 30]. This was evident for rapeseed oils tested in this study. The USFA content ranged from 84.2% to 93.9%, where significant variations were observed in the USFA subgroups MUFA and PUFA, as outlined in Table 2.

#### 3.4.1. Monounsaturated Fatty Acids (MUFA)

Due to a desirable USFA content, rapeseed oil is one of the most consumed oils due to this culinary oil's dominant MUFA content [15]. Of the rapeseed oils tested, the MUFA content consisted primarily of oleic acid (C18:1), linoleic acid (C18:2), linolenic acid (C18:3) and gadoleic acid (C20:0). Oleic acid (C18:1) is the most dominant fatty acid, corresponding with the data reported from the various studies [14, 15, 31]. MUFA content of Irish rapeseed oils varied from 40.95% to 82.38%, with oleic acid (C18:1) content ranging from 34.17% to 66.03% (Table 1), corresponding with the data reported in various studies [32–34]. The oleic acid (C18:1) content variation was considered a statistically significant difference (*p* = 0.05). The rapeseed oil from producer 4, B1, had a lower oleic acid (C18:1) content than the other Irish rapeseed oils tested. It contains lower oleic acid (C18:1) content (45.16%) and has higher SFA (11.27%), which is comparable with data reported on rapeseed oils' oxidation process [35]. Todorov reported variance in the fatty acid content of the same variant of rapeseed crops treated with different fertilisers during cultivation. It was reported that the fatty acid composition, specifically the USFA content, can be directly influenced by cultivation practices resulting in increased USFA composition. Todorov reported USFA content of the same variant of rapeseed ranging from 93.77% to 95.25% [7]. This may be an insight into why the MUFA content of the Irish rapeseed oils differ.

#### 3.4.2. Polyunsaturated Fatty Acids (PUFA)

PUFA content ranged from 9.14% to 50.69% in the Irish rapeseed oil samples tested, which was considered a statistically significant difference (*p* = 0.001). Linoleic acid (C18:2) and *α*-linolenic acid (C18:3) were the main compositional PUFAs detected, ranging from 13.00% to 40.99% and 7.85% to 14.78%, respectively, as per Table 2. The varying linoleic acid (C18:2) and linolenic acid (C18:3) content compared to data reported by other studies where the linoleic acid and linolenic acid content reported in the studies ranged from 20.24% to 2.65% and 8.71% to 9.56%, respectively [22, 24, 35]. Some of the Irish rapeseed oils contained considerably higher linoleic acid and linolenic acid content than the rapeseed oils of Greek, Slovakian and Turkish origin. The variation in linoleic acid (C18:2) content was considered statistically significant, while the differences in linolenic acid (C18:3) content were not considered statistically different (*p* < 0.05). Hernández et al. reported that fatty acid desaturase could be responsible for variations in fatty acid composition. Hernández et al. suggested that when environmental temperatures are lower than average, the PUFA content can increase in plants and, therefore, in the seeds' oils. Thus, this could be why the linoleic acid (C18:2) content of Irish rapeseed oils was higher than the earlier report [36].

PUFAs are essential fatty acids to have in culinary oils as the health impact of the oil increases with these essential fatty acids. Linoleic acid (C18:2) is a precursor for arachidic acid, a precursor for eicosapentaenoic acid (EPA). While linolenic acid (C18:3) is a precursor for EPA and docosahexaenoic acid (DHA). EPA and DHA are long-chain PUFAs, synthesised from *α*-linolenic acid (C18:3), associated with lowering cardiovascular illnesses, arthritis and inflammatory diseases[15]. Thus, the increased content of PUFA in the Irish rapeseed oil provides the desired health characteristic associated with consuming these culinary oils.

### 3.5. Variance in Batch Fatty Acids

**Figure 3:**
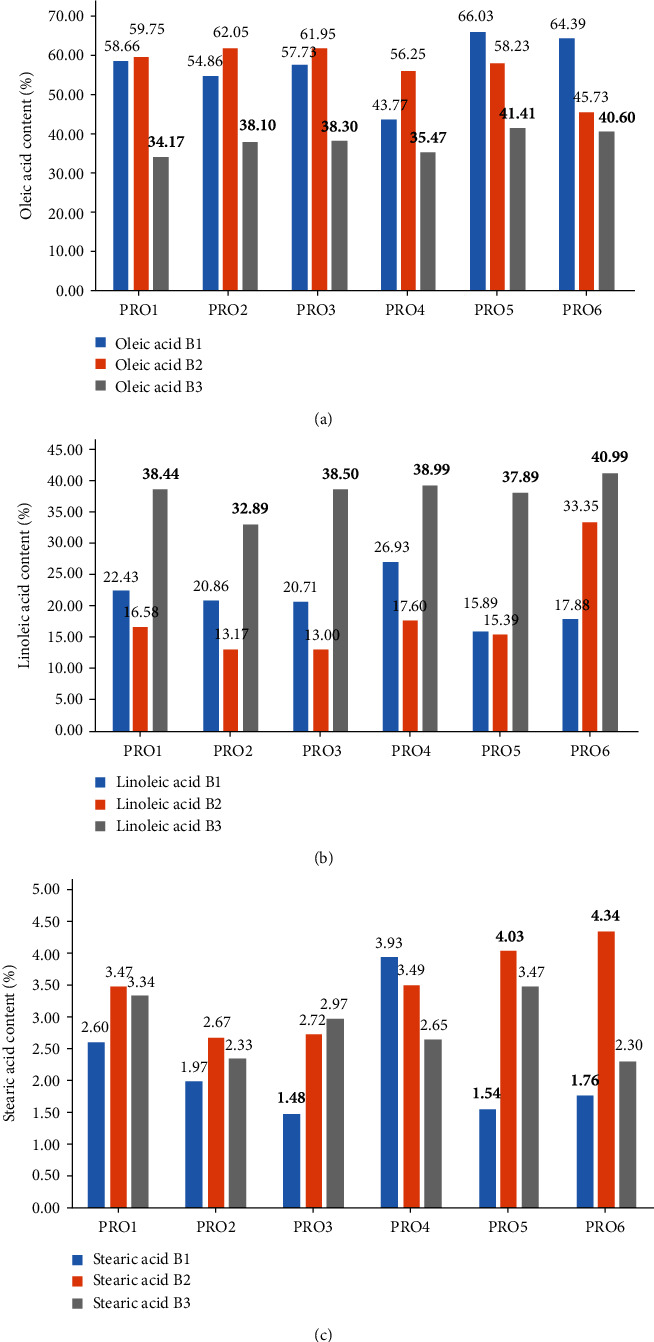
The statistical differences observed for oleic acid (C18:1), linoleic acid (C18:2), and stearic acid (C18:0) within successive producer batches (*p* ≤ 0.05): (a) oleic acid variation within batches, (b) linoleic acid variation within batches, and (c) stearic acid variation within batches.

Differences between the producer batches were evident in this study also. Oleic acid (C18:1) and linoleic acid (C18:2) demonstrated significant differences within the successive batches. Batch 3 samples demonstrated less oleic acid (C18:1) and more or equal linoleic acid (C18:2) than batches 1 & 2. ANOVA statistical analysis proved this a significant difference (where *p* ≤ 0.05). Additionally, stearic acid (C18:0) content proved to be statistically different within successive batches also. The differences observed in Table 2 for palmitic acid (C16:0), linolenic acid (C18:3), gadoleic acid (C20:0), and trace fatty acids within successive batches were not considered to be statistically different. A further inspection into the individual fatty acid content may highlight and explain these variations.  Figure 3 outlines the statistical differences observed for Oleic acid, linoleic acid and stearic acid within successive batches.

Oleic acid content ranged from 34.17% to 66.03%, linoleic acid content ranged from 13.00% to 40.99%, and stearic acid content ranged from 1.48% to 4.34% for all samples tested. Oleic acid is more heat-stable than other MUFA, making a high oleic acid containing culinary oils desirable in terms of nutrition and stability during storage and cooking [4]. As observed in Figure 3(a), the third successive batch from each producer exhibits significantly lower oleic acid content (%). While in Figure 3(b), the third batch demonstrates a significantly higher linoleic acid content (%). It should be noted that the lower oleic acid content and higher linoleic acid content balance the overall USFA content of the rapeseed oil are maintaining a desirable high USFA content.  Figure 3(c) depicts the variation of the SFA stearic acid within batches. No trend can be observed within batch lots, but significant differences can be observed. All significant differences in Figure 3 are highlighted in bold font.

### 3.6. Variation Comparison

**Figure 4:**
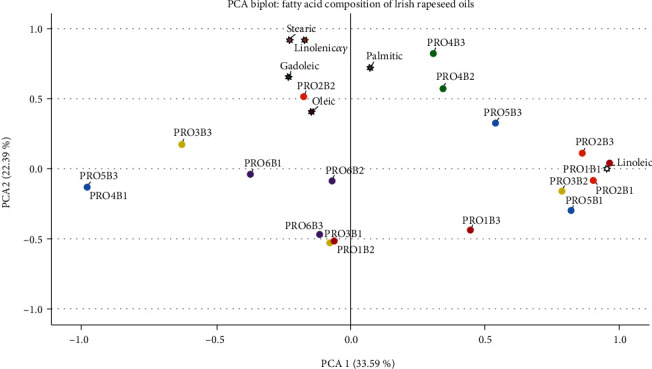
Principle Component Analysis (PCA) biplot of the fatty acid composition of rapeseed oil batches (*n* = 3) with individual fatty acids identified. PCA1 accounted for 33.59% of the variance observed in the Irish rapeseed oil batches' fatty acid profile, while PCA2 accounted for 22.39% of these variances. PRO1: red; PRO2: orange; PRO3: yellow; PRO4: green; PRO5: blue; PRO6: purple.

Figure 4 depicts the biplot used to illustrate batch variance based on individual fatty acid content. This Principle Component Analysis (PCA) determined which Irish rapeseed oil batches differentiated from each batch. Two principal components were extracted based on data variance ranging from -1 to +1. Samples were colour coded per producer, where each producer is represented by 3, one dots for each successive batch (*n* = 3). Rapeseed oils exhibited similar fatty acid trends grouped in the same component matrix as they were correlated. If the fatty acid content of one oil increased or decreased, the content of the other rapeseed oil exhibited similar trends. Regarding the PCA model, variables positioned close to the component origin (0.0) had a weaker impact on the overall model. Meaning they did not exhibit significant differences within the model trend.

PCA1 contained samples differentiated by palmitic acid (C16:0), the rapeseed oils from producer 4 are distinguishable in this component. These batch samples are positioned on opposite ends of the plot origin, which indicates a negative relationship. The rapeseed oil from producer 4, batch 1 (PRO3B1), had a significantly higher palmitic acid (C16:0) content than batches 2 or 3; therefore, it is justifiable for these samples to be on the opposite ends of the PCA 1 axis. Two rapeseed oils from producer 5, batch 2 and batch 3 (PRO5B2, PRO5B3), exhibited a similar relationship. While batch one from producer 5 lies within PCA2, indicating the fatty acid content of the rapeseed oil from PRO5B1 differed from the other batches from producer 5. Based on the data in Table 2, PRO5B1 contained less SFA content and higher USFA content than batch 2 and batch 3, which may rationalise why this sample lies in PCA2. While the oils from PRO6B1-B3, PRO3B1, and PRO1B2 distribute through PCA1, they did not contribute significantly to the model as they lie close to the origin. The samples PRO6B3, PRO3B1, and PRO1B2 lie close together in PCA1, indicating that these rapeseed oils exhibited similar variation patterns in the PCA model and, therefore, similar fatty acid content.

PCA2 distribution demonstrated variations of fatty acid content based on USFA. PCA2 contained samples differentiated by stearic acid (C18:0), oleic acid (C18:1), linolenic acid (C18:3), and gadoleic acid (C20:0) on one end of the axis. In contrast, linoleic acid (C18:2) resided at the opposite end, indicating a correlation between these variables. Linoleic acid (C18:2) was positioned close to +1 on the PCA2 axis, indicating that the fatty acid profile of the rapeseed oils in PCA2 is explained more by differences in linoleic acid (C18:2) content. The rapeseed oil from producer 2, batch 2 (PRO2B2), was positioned closer to oleic acid (C18:1) due to the higher oleic acid (C18:1) content this rapeseed oil contained. In contrast, batch 1 from producer 2 (PRO2B1) had a higher linoleic acid (C18:2) content than PRO2B2, which justifies the position of this sample in the PCA plot; these batches were inversely proportional in the model. Batch 3 from producer 2 (PRO2B3) was also positioned in PCA2; however, this batch aligned closer to the PCA1 axis. While this rapeseed oil exhibited a low content of palmitic acid (C16:0), it also contained higher linoleic acid (C18:2) than both batches. Hence, this sample contributed variance in the model's palmitic acid (C16:0) and linoleic acid (C18:2) content.

The position of PRO3B2 and PRO3B3 in PCA2 demonstrates different linoleic acid (C18:2) and oleic acid (C18:1) content, resulting in these samples being distributed on opposite ends PCA2 axis. PRO3B2 had significantly less oleic acid (C18:1) content than PRO3B3 but presented a considerably higher linoleic acid (C18:2) content, as shown in Figure 4.

Based on the findings in this study outlined in Table 2 and confirmed in Figures 3 and 4, rapeseed oils from Irish producers possess high concentrations of desirable fatty acid such as oleic acid (C18:1), linoleic acid (C18:2) and linolenic acid (C18:3) which confirms that rapeseed oils produced in Ireland have a nutritionally beneficial fatty acid composition which can contribute to consumer health. Additionally, this study highlighted variations in rapeseed oils' fatty acid composition from 6 different Irish producers. Furthermore, these variations can be observed for individual fatty acids of the 3 successive batches per producer.

## 4. Conclusion

The fatty acid content of Irish rapeseed oils was nutritionally beneficial due to the high unsaturated fatty acid proportion of the overall profile and the desirable MUFA and PUFA content these oils possess, which can contribute to consumers health. The fatty acid composition demonstrated significant differences in individual fatty acid content, while total saturation had insignificant differences. The reason for the variation in individual fatty acids of the rapeseed oils tested was unknown. However, as previously mentioned, many studies have contributed cultivation practices, improper processing, harvesting time and geographical regions as influential factors to an oil's fatty acid content.

## Figures and Tables

**Table 1:** Database of fatty acid information used to analyse chromatograms. The theoretical m/*z* of individual fatty acids and the experimental m/*z* detected of the individual fatty acids.

Fatty acid	Molecular formula	C : U	Molecular mass (g/Mol)	m/*z* (M-H)^−^ theoretical	m/*z* (M-H)^−^ experimental
Caprylic	C8 H16 O2	08 : 00	144.12	*143.108*	143.108
Pelargonic	C9 H18 O2	09 : 00	158.13	*157.123*	157.123
Capric	C10 H20 O2	10 : 00	172.15	*171.139*	*n.d*
Undecylic	C11 H22 O2	11 : 00	186.16	*185.155*	*n.d*
Lauric	C12 H24 O2	12 : 00	200.18	*199.17*	199.170
Tridecylic	C13 H26 O2	13 : 00	214.19	*213.186*	*n.d*
Myristic	C14 H28 O2	14 : 00	228.21	*227.202*	227.202
Pentadecylic	C15 H30 O2	15 : 00	242.22	*241.217*	241.217
Palmitic	C16 H32 O2	16 : 00	256.24	*255.233*	255.233
Palmitoleic	C16 H30 O2	16 : 01	254.22	*253.217*	253.217
Palmitelaidic	C16 H30 O2	16 : 01	254.22	*253.217*	*n.d*
Margaric	C17 H34 O2	17 : 00	270.26	*269.249*	269.249
Stearic	C18 H36 O2	18 : 00	284.27	*283.264*	283.264
Oleic	C18 H34 O2	18 : 01	282.26	*281.249*	281.249
Elaidic	C18 H34 O2	18 : 01	282.26	*281.249*	*n.d*
Linoleic	C18 H32 O2	18 : 02	280.24	*279.233*	279.233
Linolenic (*α*)	C18 H30 O2	18 : 03	278.22	*277.217*	277.217
Stearidonic	C18 H28 O2	18 : 04	276.21	*275.202*	275.202
Nonadecylic	C19 H38 O2	19 : 00	298.29	*297.28*	*n.d*
Arachidic	C20 H40 O2	20 : 00	312.3	*311.296*	311.296
Gadoleic	C20 H38 O2	20 : 01	310.29	*309.28*	309.287
Gondoic	C20 H38 O2	20 : 01	310.29	*309.28*	
Dihomolinoleic	C20 H36 O2	20 : 02	308.27	*307.264*	307.264
Dihomolinolenic	C20 H34 O2	20 : 03	306.26	*305.249*	305.249
Mead acid	C20 H34 O2	20 : 03	306.26	*305.249*	*n.d*
Arachidonic	C20 H32 O2	20 : 04	304.24	*303.233*	303.233
Eicosatetraenoic	C20 H32 O2	20 : 04	304.24	*303.233*	*n.d*
EPA	C20 H30 O2	20 : 05	302.25	*301.217*	*n.d*
Heneicosylic	C21 H42 O2	21 : 00	326.32	*325.311*	*n.d*
Behenic	C22 H44 O2	22 : 00	340.33	*339.327*	339.320
Eruic	C22 H42 O2	22 : 01	338.32	*337.311*	337.311
Docosadienoic	C22 H40 O2	22 : 02	336.3	*335.296*	335.296
Eranthic	C22 H38 O2	22 : 03	334.29	*333.28*	*n.d*
Ardenic	C22 H36 O2	22 : 04	332.27	*331.264*	*n.d*
DPA	C22 H34 O2	22 : 05	330.26	*329.249*	*n.d*
DHA	C22 H32 O2	22 : 06	328.24	*327.233*	*n.d*
Tricosylic	C23 H46 O2	23 : 00	354.35	*353.343*	*n.d*
Lignoceric	C24 H48 O2	24 : 00	368.37	*367.358*	367.358

**Table 2:** Fatty acid profile of rapeseed oil batches (*n* = 3) outlining the primary compositional fatty acids.

Saturated fatty acids (%)					Unsaturated fatty acids (%)					
Producers	Palmitic	Stearic	Other	Total SFA	Oleic	Linoleic	Linolenic (*α*)	Gadoleic	Other	Total USFA
PRO1B1	3.12	2.60	1.91	*7.6*	58.66	22.43	8.26	2.85	0.19	*92.4*
PRO1B2	2.67	3.47	0.73	*6.9*	59.75	16.58	14.50	2.03	0.26	*93.1*
PRO1B3	4.41	3.34	1.44	*9.2*	34.17^∗^	38.44^∗^	14.13	3.46	0.62	*90.8*
PRO2B1	4.01	1.97	0.70	*6.7*	54.86	20.86	11.05	3.66	2.88	*93.3*
PRO2B2	2.21	2.67	1.26	*6.1*	62.05	13.17	9.59	4.74	4.32	*93.9*
PRO2B3	2.53	2.33	1.99	*6.8*	38.10^∗^	32.89^∗^	14.31	3.09	4.75	*93.2*
PRO3B1	5.75	1.48	0.99	*8.2*	57.73	20.71	10.90	2.16	0.28	*91.8*
PRO3B2	2.76	2.72	0.82	*6.3*	61.95	13.00	14.78	3.75	0.23	*93.7*
PRO3B3	2.96	2.97	1.94	*7.9*	38.30^∗^	38.50^∗^	13.54	1.47	0.33	*92.1*
PRO4B1	7.99	3.93	3.85	*15.8* ^∗^	43.77	26.93	8.17	4.35	1.01	*84.2* ^∗^
PRO4B2	3.89	3.49	1.80	*9.2*	56.25	17.60	12.70	3.89	0.36	*90.8*
PRO4B3	3.67	2.65	2.55	*8.9*	35.47^∗^	38.99^∗^	13.43	2.89	0.36	*91.1*
PRO5B1	4.43	1.54	0.84	*6.8*	66.03	15.89	9.99	1.05	0.22	*93.2*
PRO5B2	3.62	4.03	1.97	*9.6*	58.23	15.39	13.95	2.47	0.35	*90.4*
PRO5B3	3.51	3.47	2.34	*9.3*	41.41^∗^	37.89^∗^	9.97	1.11	0.30	*90.7*
PRO6B1	4.24	1.76	1.16	*7.2*	64.39	17.88	9.26	1.13	0.19	*92.8*
PRO6B2	4.49	4.34	1.07	*9.9*	45.73	33.35	7.85	3.12	0.04	*90.1*
PRO6B3	3.69	2.30	1.40	*7.4*	40.60^∗^	40.99^∗^	10.16	0.53	0.33	*92.6*

∗Significantly different (*p* < 0.05).

## Data Availability

The data that support the findings of this study are available from the corresponding author upon reasonable request.

## Abbreviations

SFA: Saturated fatty acids
USFA: Unsaturated fatty acids
MUFA: Monounsaturated fatty acids
PUFA: Polyunsaturated fatty acids
LCMS: Liquid chromatography-mass spectroscopy
LDL: Low-density lipoprotein
HDL: High-density lipoprotein.
